# Evaluation of the Antiradical Activity of *Schisandra Chinensis* Lignans Using Different Experimental Models

**DOI:** 10.3390/molecules15031223

**Published:** 2010-03-03

**Authors:** Karel Šmejkal, Tereza Šlapetová, Pavel Krmenčík, Renata Kubínová, Pavel Suchý, Stefano Dall´Acqua, Gabbriella Innocenti, Ján Vančo, Karolína Kalvarová, Margita Dvorská, Jiří Slanina, Eva Kramářová, Jan Muselík, Milan Žemlička

**Affiliations:** 1 Department of Natural Drugs, Faculty of Pharmacy, University of Veterinary and Pharmaceutical Sciences Brno, Brno, Czech Republic; 2 Department of Human Pharmacology and Toxicology, Faculty of Pharmacy, University of Veterinary and Pharmaceutical Sciences Brno, Brno, Czech Republic; 3 Department of Pharmaceutical Sciences, University of Padua, Padua, Italy; 4 Department of Chemical Drugs, Faculty of Pharmacy, University of Veterinary and Pharmaceutical Sciences Brno, Brno, Czech Republic; 5 Department of Biochemistry, Faculty of Medicine, Masaryk University, Brno, Czech Republic; 6 Department of Pharmaceutics, Faculty of Pharmacy, University of Veterinary and Pharmaceutical Sciences Brno, Brno, Czech Republic

**Keywords:** *Schisandra chinensis*, dibenzocyclooctadiene lignans, antiradical, diabetes mellitus

## Abstract

The *in vitro* antiradical activity of *Schisandra chinensis* lignans was investigated using DPPH, ABTS^+^, Fenton reaction inhibition and tyrosine-nitration inhibition assays, as were the *in vivo* antidiabetic activities of selected lignans in an animal model of alloxan-induced diabetes. Different degrees of antiradical activity were found, depending upon the structural parameters of the tested compounds. Unfortunately, the compounds showed no antidiabetic activity in concentration range tested.

## 1. Introduction

Dibenzocyclooctadiene lignans were found to be the main group of compounds contained in the fruit of *Schisandra chinensis* and to date more than 40 dibenzocyclooctadiene lignans have been isolated from this source [1]. Attention has been focused on this plant because of its use in traditional Chinese herbal therapy, where the fruit of *S. chinensis* is highly valued, especially for its effects as a stimulant and anti-aging agent. Extracts or pure compounds of *Schisandra* are well known to have therapeutic effects, serving as tonics and hepatoprotective agents. Tests of pure isolated compounds have identified adaptogenic, antioxidative, hepatoprotective and cardioprotective activities [2,3,4,5].

The prevalence of diabetes mellitus, a chronic metabolic disorder found in approximately 5% of the population of industrialized countries, is increasing rapidly. Diabetes mellitus type 1 is connected with hypoinsulinaemia and the destruction of the islets of Langerhans in the pancreas. The cause of this deterioration remains unclear, although several hypotheses have been advanced, including an autoimmune response of the organism, viral attack, or the toxic influence of certain chemicals. Increased levels of reactive oxygen and nitrogen species (ROS, RNS) in the diabetic pancreas have been clearly documented. Alloxan (pyrimidine-2,4,5,6-tetraone), a frequently used diabetogenic chemical, shows selective toxicity to β-cells of the islets of Langerhans. Its diabetogenic action is mediated by inhibiting the enzyme glucokinase through oxidation of the two thiol groups at the glucose-binding site of the enzyme [6,7]. Its cytotoxic effect on β-cells seems to be associated with the cyclic redox process of the alloxan-dialuric acid interconversion, producing ROS, i.e. a superoxide radical anion, hydrogen peroxide, and a hydroxyl radical [8,9]. Due to this effect, alloxan can be used to generate a pathophysiological status, similar to Type 1 diabetes. In addition, the low expression of antioxidant enzymes by these tissues relative to other tissues leaves β-cells particularly vulnerable to the effects of ROS [10,11], a very important factor in the development of diabetes.

Previous studies have shown that some plant extracts and natural compounds posses a protective effect against alloxan-induced diabetes. Natural polyhydroxylated compounds show such activity, possibly due to their antioxidative properties [12,13,14]. The positive influence of dibenzocyclooctadiene lignans on antioxidant biological systems has been described previously [2]. The effect on glutathione levels and antioxidative activities, including the induction of antioxidative enzymes, inhibition of CCl_4_-induced lipoperoxidation, and the enhancement of the hepatic antioxidant system have also been described [2,3]. Therefore, the presence of a range of related dibenzocyclooctadiene lignans in *S. chinensis* makes this species an interesting candidate to investigate for antidiabetic activity. Many different assays are used for testing the antiradical activity of potential pancreas-protective therapeutics, usually *in vitro*. The DPPH^•^, ABTS^•+^ and the inhibition of the Fenton reaction assays are widely used [15,16,17]. Our principal aim was to evaluate the antiradical activity of lignans with these commonly used tests in addition to the inhibition of a peroxynitrite-mediated nitration of tyrosine. Furthermore, we focused on testing the purified compounds for their antidiabetic activity against alloxan-induced diabetes using an *in vivo* assay.

## 2. Results and Discussion

**Figure 1:**
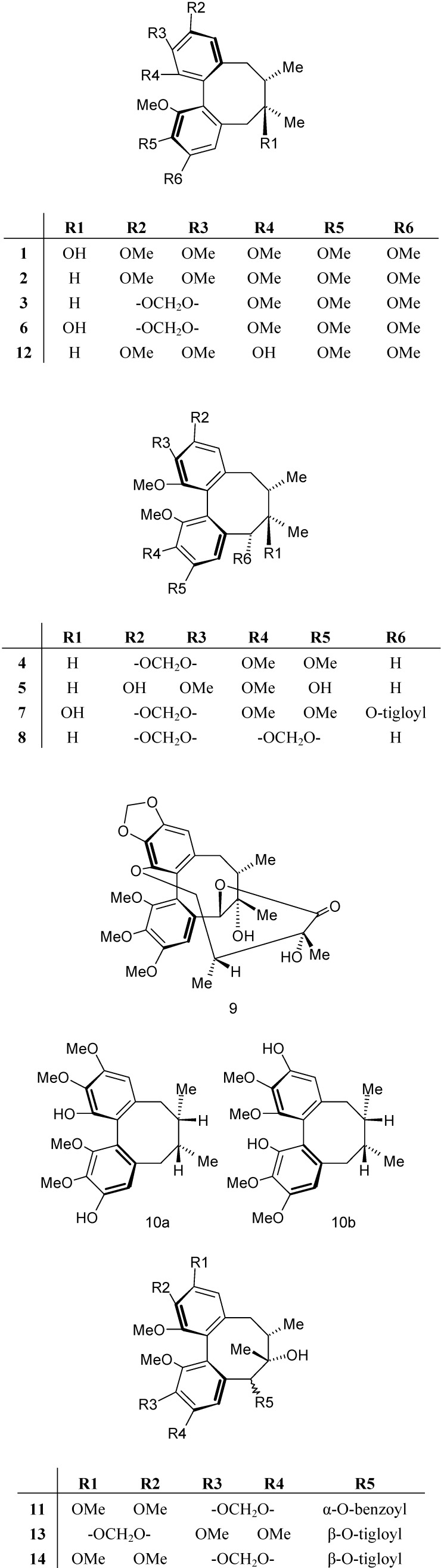
Studied lignans.

Our investigation of the phytoconstituents of the fruit of *S. chinensis* led to the isolation and structural determination of 14 lignans (Figure 1): (+)-schisandrin (**1**), (+)-deoxyschisandrin (**2**), (+)-γ-schisandrin (**3**), (-)-gomisin N (**4**), (-)-gomisin J (**5**), (+)-gomisin A (**6**), (-)-tigloyl-gomisin P (**7**), (-)-wuweizisu C (**8**), (-)-gomisin D (**9**), rubrisandrin A (**10**) (an inseparable mixture of regioisomers 10a and 10b in a 2:1 ratio), (-)-gomisin G (**11**), (+)-gomisin K_3_ (**12**), (-)-schisantherin C (**13**), and (-)-tigloyldeangeloyl-gomisin F (**14**).

The antiradical activity was tested using the DPPH^•^, ABTS^•+^, Fenton reaction, and tyrosine-nitration inhibition methods. The DPPH assay showed very weak activity when results were displayed as Trolox equivalents (Table 1). These results are consistent with the previously published hypothesis that only compounds able to donate a proton and an electron and to form stable structures are good scavengers of DPPH [18]. The *S. chinensis* lignans **1**, **2**, **6**–**8**, **11**, **13** and **14** do not have free phenolic OH groups which could donate protons. The activity of **5**, **10**, and **12** is only slightly higher, because the phenolic hydroxyl is probably sterically blocked. Surprisingly, (-)-gomisin D (**9**), which shows the highest DPPH scavenging activity, also has no free phenolic OH group. Its structure is unusual due to the presence of an additional nine-membered ring arising from the cyclization of the tigloyl moiety with a phenolic hydroxyl group. Similar results were obtained with the ABTS^•+^ assay. This assay is based on same principle as DPPH^•^ scavenging: compounds that can donate a proton react as scavengers. Compounds **1**–**14** were found to be very weak scavengers of ABTS^+^ radicals as compared with Trolox.

**Table 1:** Antioxidative activities of *S. chinensis* lignans **1**–**14** found using four experimental methods. Results are expressed as Trolox Equivalent Antioxidant Capacity (TEAC).

Compound	Antioxidative activity expressed as TEAC			
	DPPH^•^	ABTS^•+^	Fenton reaction inhibition	Tyrosine nitration inhibition
**1**	0.0108	0.0038	0.0060	0.016
**2**	0.0067	0.0044	-^a^	0.047
**3**	0.0083	0.0095	0.0179	0.048
**4**	0.0025	-^a^	0.0095	0.017
**5**	0.0160	0.0214	0.0145	0.765
**6**	0.0040	0.0031	0.0090	0.016
**7**	0.0046	0.0152	0.0158	-^a^
**8**	0.0036	0.0035	0.0083	0.027
**9**	0.1096	-^b^	0.0159	0.791
**10**	-^b^	-^b^	-^b^	0.046
**11**	0.0073	-^b^	0.0170	0.132
**12**	0.0739	-^b^	0.0748	0.315
**13**	0.0002	-^b^	0.0115	-^a^
**14**	-^a^	-^b^	0.0134	-^b^

^a^ No measurable activity, ^b^ not determined.

Compounds **1**–**14** were also tested for their ability to inhibit Fe^2+^/H_2_O_2_–dependent chemiluminescence. In this assay, a hydroxyl radical generated by the Fenton reaction oxidizes luminol, which induces the emission of light (λ 430 nm) upon decomposition. The highest activity was shown by gomisin K_3_ (**12**). On the other hand, almost zero activity was found for (+)-schisandrin (**1**) or (+)-deoxyschisandrin (**2**). Other compounds tested showed moderate activity, with values ranging from 0.083 to 0.0179. As can be seen in Table 1, all the compounds tested were found to be weak OH^•^ scavengers when compared with Trolox. The observed activity is probably connected more with the scavenging of the hydroxyl radical than with the ability to chelate Fe^2+^.

The evaluation of tyrosine-nitration inhibition showed some different results. Compounds **1**–**4**, **6**–**8**, **10** and **13** were found to be very weak scavengers, compared to Trolox. Compounds **11** and **12** showed moderate activity, with TEAC values of 0.132 and 0.315, respectively. Only compounds **5** and **9** showed considerable ability to inhibit tyrosine nitration; respective TEAC values of 0.765 and 0.791 were calculated for them. This correlates partly with the other results obtained.

The ability of pure *S. chinensis* lignans **1**–**8** to prevent alloxan-induced pancreatic damage *in vivo* was tested in laboratory mice. Changes in weight and glycaemia level were monitored. Unfortunately, at the concentration used (100 μmol/kg), no statistically important differences were observed when compared with experimental controls (data not shown).

These antiradical assays show that contrary to the reports in literature, *Schisandra* lignans are not good direct scavengers of free radicals. When pure lignans were previously tested, the greatest activity was most often found for gomisin K_3_ (**12**) [3,5], which corresponds with our findings. On the other hand, γ-schisandrin (**3**), presented in the literature as a good stimulant of the antioxidant defensive response [2,4], showed little ability to scavenge hydroxyl radical, peroxynitrite products or synthetic radicals directly. It should be mentioned that the previously published studies on the direct antiradical activity of *Schisandra* deal mainly with methanolic or ethanolic extracts, and not with the pure isolated compounds [19,20,21]. Some of the effects of the structure on the activity of pure lignans were carried out. The effect of the structure on the antioxidant activity in the DCFH-DA cellular based assay system revealed the importance of the exocyclic methylene group, the benzoyl moiety and the angeloyl substitution of the aromatic rings for the antioxidant effect [22]. In our assays, compounds did not fulfill these previously stated conditions, only gomisin G (**11**) with benzoyl moiety showed some activity in tyrosine-nitration inhibition assay.

Hence it can be concluded that the *Schisandra* lignans alone are not fully responsible for the previously found antiradical activity and that other compounds present in *S. chinensis* must contribute to the effects described. This does not mean that dibenzocyclooctadiene lignans have no effect on the anti-oxidative defense. The antioxidative activity of dibenzocyclooctadiene lignans is connected with the level of glutathione, the activity of glutathione peroxidase and the inhibition of the formation of cellular peroxides [23]. The effects of lignans on the antioxidative enzymatic system of the liver have been previously described and reviewed [3,5]. According to these reports, the mechanism of the antiradical activity of *Schisandra* lignans should be studied *in vivo*. We decided to prove previous results by assaying lignans using an alloxan-induced diabetes test in mice. We found no activity at the concentration tested. This partially refutes the reports of a chemoprotective effect of *S. chinensis* lignans on liver tissue [24], but it may be due to the major differences between the metabolic activity and enzymatic apparatus of the pancreas and the liver [9,10].

## 3. Experimental

### 3.1. Plant material

The fruits of *S. chinensis* (produced in China) were purchased from Ing. Adolf Pleskač, Rosa Canina Ltd. (Czech Republic) in 2006. The material was identified by Assoc. Prof. Petr Babula of the Department of Natural Drugs, Faculty of Pharmacy, University of Veterinary and Pharmaceutical Sciences Brno, Brno, Czech Republic. A voucher specimen (SC-2006) was deposited in the herbarium of Department of Natural Drugs.

### 3.2. Extraction and isolation

One kilogram of *S. chinensis* fruits was lyophilized and extracted using maceration in MeOH. Dibenzocyclooctadiene lignans **1**–**14** were isolated by preparative and semipreparative HPLC (data not shown). The purity of compounds isolated exceeded 95% according to the HPLC-DAD evaluation.

### 3.3. In vitro DPPH^•^, ABTS^•+^ and chemiluminescence inhibition assays

Assays were performed on a Synergy^HT^ multiplate reader (Biotek, USA). For DPPH^•^ and chemiluminescence inhibition, solutions of the samples were prepared in MeOH; for ABTS^•+^ they were made in EtOH. 96-well plates were used. Calibration curves prepared using measurements of Trolox at six different concentrations were used to establish the TEACs. The hydroxyl radical scavenging activity was measured by monitoring the hydroxyl radical-induced oxidation of luminol, accordingly to a version of the slightly modified procedure presented in [16]. Hydroxyl radical was generated using a Fenton system (Fe^2+^/H_2_O_2_). The reaction mixtures in the sample wells contained the following reagents: FeSO_4_ (25 μM, 30 μL), H_2_O_2_ (0.5 mM, 50 μL), and the sample tested (10 μL), to be total volume adjusted with H_2_O to 330 μL. The reaction was initiated by adding 1 mM luminol in 0.1M NaOH (100 μL). Dynamic curves were recorded after the initiation of reaction using a microplate reader for 30 minutes in minute interval. The scavenging effect (%) was calculated according to the formula:

Scavenging effect [%] = 1 - (Sample AUC/Control AUC) × 100.

### 3.4. Inhibition of tyrosine-nitration assay

This assay was performed using the routine procedure on an Agilent 1100 DAD apparatus detailed in reference [17]. Samples for measurement were prepared by dissolving pure lignans in 2-methoxy-ethanol.

### 3.5. Cytoprotective effect against alloxan-induced diabetes in vivo

Female ICR (imprinting control region) albino mice (30–40 g; Anlab, Czech Republic) were used in the experiment. The mice were divided into 10 experimental groups: eight of these were pretreated with the tested compounds **1**-**8**, one was a positive control (only alloxan solution administered) and one was a negative control (only isotonic saline solution was administered). Each group consisted of ten mice (n = 10). The compounds to be tested (**1**–**10**) and alloxan monohydrate were dissolved in 10% DMSO in isotonic saline solution (v/v). The solutions of the test compounds were administered intraperitoneally (0.1 mL/10 g body weight, at doses of 100 μmol/kg of body weight). The alloxan solution was injected into the tail vein (0.1 mL/10 g body weight, at doses of 100 mg/kg of body weight) 30 min after the application of the test compound. The initial glucose levels were measured in the intact mice before applications. During the next 4 days of the experiment the glucose levels were monitored in the morning after at least 3 h of fasting (day 2–5). One-drop glucose oxidase test and blood glucose reflective photometer Glucotrend® 2 with Glucotrend® Glucose (max. concentration 33.3 mM; Roche, Germany) test strips were used to determine glucose concentrations (mM) in the venous blood. The observed changes in glucose levels were evaluated with the ANOVA method. All aspects of the animal care complied with ethical guidelines, and the technical requirements were approved as consistent with the Animal Scientific Procedures Act 86/609/EC.

## 4. Conclusions

Fourteen lignans obtained from *S. chinensis* were tested for their potential ability to scavenge free radicals. Four different *in vitro* assays showed that their potential to scavenge free radicals directly is low; however gomisin J (**5**) and gomisin D (**9**) showed significant activity in the inhibition of tyrosine-nitration assay. Based on the previous results we examined the ability of lignans to prevent alloxan-induced diabetes in mice (based on radical damage), but the lignans we tested showed no activity at the concentrations tested.
